# Identification and Pathogenicity of *Fusarium* Species Associated with Onion Basal Rot in the Moscow Region of Russian Federation

**DOI:** 10.3390/jof10050331

**Published:** 2024-05-04

**Authors:** Svetlana Vetrova, Ksenia Alyokhina, Irina Engalycheva, Elena Kozar, Kseniya Mukhina, Maria Sletova, Leonid Krivenkov, Tatyana Tikhonova, Alina Kameneva, Svetlana Frolova, Vera Chizhik, Viktor Martynov

**Affiliations:** 1 Federal State Budgetary Scientific Institution Federal Scientific Vegetable Center, 143072 Moscow, Russia; xenia.aliokhina@yandex.ru (K.A.); engirina1980@mail.ru (I.E.); kozar_eg@mail.ru (E.K.); kseniyamukhina@yandex.ru (K.M.); gvina@yandex.ru (M.S.); krivenkov76@mail.ru (L.K.); tikhonova@tatyana94.ru (T.T.); alina.malina1290@gmail.com (A.K.); svetlanaleonidovna95@gmail.com (S.F.); chizhikvera@bk.ru (V.C.); 2 Federal State Budgetary Scientific Institution All-Russian Research Institute of Agricultural Biotechnology, 127550 Moscow, Russia; martynov.vik@gmail.com

**Keywords:** *Fusarium*, *Allium cepa* L., morphology, pathogenicity, phylogenetic analysis

## Abstract

Fusarium basal rot of onions causes large losses during storage of commercial production of onion bulbs, which in turn adversely affects the food market situation in the off-season period. There are no data on the composition of *Fusarium* spp., which causes onion basal rot in the Russian Federation. Therefore, our research was aimed at *Fusarium* spp. causing onion basal rot in the Moscow Region of the Russian Federation and studying the pathogenicity of these species for the host plant. We studied 20 isolates of *Fusarium* spp. collected from affected mature bulbs and seed bulbs. Species identification of the isolates was carried out using analysis of the nucleotide sequences of the three genetic loci ITS, *tef1* and *rpb2*, as well as was based on the macro- and micromorphological characteristics of these isolates. As a result, the species *F. annulatum* (*F. fujikuroi* species complex), *F. oxysporum* (*F. oxysporum* species complex), *F. acuminatum* (*F. tricinctum* species complex) and *F. solani* (*F. solani* species complex) were identified to involve in the pathogenesis of Fusarium basal rot. We have shown for the first time that the species *F. annulatum* and *F. acuminatum* are highly aggressive and capable of causing onion basal rot. The predominant species were *F. annulatum* and *F. oxysporum*. The proportion of these species in the total number of analyzed isolates was 60% and 25%, respectively. The largest proportion (33%) of highly aggressive on mature bulbs isolates was found in the species *F. annulatum*. The data obtained provide practical insights for developing strategies to manage *Fusarium* fungi responsible for onion basal rot Moscow Region of the Russian Federation. In addition, data about species composition and aggressive isolates may be used in onion breeding for resistance to Fusarium basal rot.

## 1. Introduction

Bulb onion (*Allium cepa* L.*)* is one of the most marketable and highly-demanded vegetable crops in the Russian Federation and other countries all over the world [1,2]. This vegetable contains many valuable nutrients and is also bactericidal, suppressing the growth of human pathogens [3,4]. As a result, onion is an indispensable component of a healthy diet.

According to the latest data from FAO, global onion production in 2021 was 78.4 million tons. The area under this crop amounted to 6.8 million hectares, and the average yield was 23.9 t/ha. The largest producers of onions are India, China and Egypt [5]. In the Russian Federation in 2021, the gross yield of commercial onion bulbs amounted to 1.6 million tons. From March to July, there is a shortage of onions in the Russian Federation, which is replenished by imports from Egypt, China, Kazakhstan and other countries.

The abovementioned shortage during this period accounts for storage losses caused by fungal and bacterial diseases [1]. The most harmful and economically significant disease of onions is caused by *Fusarium* species [6]. Various *Fusarium* species are capable of affecting onion plants at different stages of their ontogenesis, causing Fusarium wilt (FW) during the growing period and Fusarium basal rot (FBR) during storage [7,8]. In the case of FBR, the affected bulb completely rots or becomes mummified. Yield losses from FBR during the growing period have been reported to range from 3% to 35% depending on environmental conditions, cultivar and pathogen load. But, the greatest damage from FBR is observed during the storage of commercial mature bulbs and seed bulbs. These losses are as big as 40% [9].

In many onion-planting countries around the world, the most common and harmful causative agent of Fusarium basal rot is *F. oxysporum* var. *cepae* [10]. Other species reported as economically important causative agents of onion basal rot are *F. proliferatum*, *F. culmorum*, *F. solani*, and *F. redolens* [11]. Recent publications describe species *F. falciforme*, *F. brachigibbosum*, *F. anthophilium* and *F. acutatum* as causative agents of diseases affecting onion plants at different stages of their development [7,12,13].

Species identification of *Fusarium* fungi by analyzing their micro-morphological and cultural characteristics is often difficult due to the high similarity of these characteristics in different species [6,14]. Molecular genetic methods are of great help in determining the species of *Fusarium* fungi. The most phylogenetically informative and frequently used markers in these methods are the nucleotide sequences of the *tef1* gene, encoding the translation elongation factor 1 alpha [15,16], *rpb2* gene, encoding the second subunit of RNA polymerase II [17], and the *CYP51C* gene, encoding the sterol-14-demethylase [18].

Currently, in the Russian Federation, there are no data on the composition of *Fusarium* spp., which causes onion basal rot. Thus, the purpose of this study was the species identification of *Fusarium* that causes onion basal rot in the Moscow Region of the Russian Federation, as well as the assessment of their pathogenicity for onion plants. The results of the research will be used in breeding onions for resistance to FBR.

## 2. Materials and Methods

The materials for research were 20 isolates of *Fusarium* fungi isolated from affected mature bulbs (13 isolates) and seed bulbs (7 isolates) in 2022–2023 years. Diseased plant materials (89 samples) were collected in experimental fields and at the vegetable storehouse of FSBSI FSVC (Moscow Region, Russia).

The isolates were grown on potato dextrose agar (PDA) medium for 14 days under alternating light conditions (16 h—light and 8 h—dark) at 25 °C. The morphology of the colonies, the appearance of pigmentation, and the growth rate of fungi were assessed daily, recording the time of the beginning of growth. The diameter of the colonies was measured in two perpendicular directions for three parallel inoculations (n = 3) until the plate was completely covered by mycelium. The microscopic characteristics of pure cultures of micromycetes were examined and recorded using a Zeiss Axio Lab A1 microscope (ZEISS, Jena, Germany) and ADF Image Capture software (version ×64, 4.11.21522.20221011). At least 30–40 microstructures (conidia, chlamydospores) were measured for each isolate. The taxonomic status of *Fusarium* fungi was determined according to [19], including those from scientific publications as well.

The air mycelium of pathogens was collected from Petri dishes (Helicon, Moscow, Russia) and moved to 1.5 mL Eppendorf tubes (Eppendorf Group, Hamburg, Germany). DNA isolation was carried out using the DNeasy Plant Pro Kit (QIAGEN, Hilden, Germany). DNA purity was assessed using a NanoDrop device (Thermo Fisher Scientific, Waltham, MA, USA), and the concentration was measured using a Qubit 4 device (Thermo Fisher Scientific, Waltham, MA, USA).

Primer sequences and PCR amplification conditions for ITS, *tef1* and *rpb2* loci were the same as described elsewhere (Table 1).

PCR mixture contained 2.5 μL of 10× PCR buffer, 1–10 ng of genomic DNA, 2.5 μL of 2.5 M dNTP, 10 pmol of each primer, 1 unit of Taq DNA polymerases or Pfu DNA polymerase (Evrogen, Moscow, Russia), and sterile water to a volume of 25 μL. Amplification was performed in an MJ PTC-200 thermal cycler (Bio-Rad, Hercules, CA, USA) using the following cycling procedure: one cycle of 94 °C for 3 min; 35 cycles of 94 °C for 30 s, Tm (Table 1) for 30 s and 72 °C for 1 min; and a final extension at 72 °C for 5 min. Nucleic acids were separated in agarose gel using electrophoresis. The horizontal electrophoresis chamber Sub-Gell GT System and the PowerPac HC power supply (BioRad, Hercules, CA, USA) were used for the analysis. Visualization of the results was performed on a GelDoc XR+ System transilluminator (Bio Rad, Hercules, CA, USA). Excision of PCR products from the gel was performed on an ECX-M transilluminator (VilberLourmat, Eberhardzell, Germany). The samples were also purified with a ColGen kit (Syntol, Moscow, Russia) and sequenced at Syntol. Species identification was carried out in GenBank NCBI [22] and FUSARIOID-ID database—Food, Fibre & Health [23,24].

Phylogenetic relationships between sequences were inferred by using the Maximum Likelihood method and the Tamura–Nei model [25]. The tree with the highest log likelihood (−9696.74) is shown. The percentage of trees in which the associated taxa clustered together is shown next to the branches. Initial tree(s) for the heuristic search were obtained automatically by applying Neighbor-Join and BioNJ algorithms to a matrix of pairwise distances estimated using the Maximum Composite Likelihood (MCL) approach and then selecting the topology with superior log likelihood value. The tree is drawn to scale, with branch lengths measured in the number of substitutions per site. This analysis involved 19 nucleotide sequences. Codon positions included were 1st + 2nd + 3rd + Noncoding. There were a total of 1745 positions in the final dataset. Evolutionary analyses were conducted in MEGA X [26].

Initially, the pathogenicity assessment of 20 *Fusarium* isolates and confirmation of Koch’s postulates for them were carried out on two widely cultivated onion varieties, «Globus» and «Atas» of the FSBSI FSVC selection. Five bulbs of each variety in one replication were used for the study. The experiments were carried out in five replications. Two outer succulent scales of sterilized bulbs were used for inoculation. These scales were cut into identical portions of 2 × 2 cm, as described by Mizue Tsuji [27], and a thin transparent film was removed from the inner scales to create a wound surface. These portions were placed into plastic boxes for subsequent inoculation. Inoculation was carried out with a 10-day agar block culture of Fusarium fungi. A sterile agar block was used as a negative control. The boxes were incubated in the dark for two days, followed by incubation under alternating light conditions (16 h—light and 8 h—dark) at 25 °C. The extent of the lesion was determined on the 10th-day post-inoculation by measuring the diameter and depth of the affected area. The volume of the affected area (Va, cm^3^) was calculated using the formula for the volume of a cylinder, measuring the radius and depth of the affected area:

$$Volume\;of\;the\;affected\;area\left(Va\right)=\pi \times {\left(radius\;of\;the\;affected\;area\right)}^{2}\times depth\;of\;the\;affected\;area$$

Based on the volume of the affected area analyzed, isolates were classified as follows:

Non-pathogenic (NP): Va = 0 mm^3^;

Weakly aggressive (WA): Va = 1–50 mm^3^;

Moderately aggressive (MA): Va = 51–150 mm^3^;

Highly aggressive (HA): Va ≥ 151 mm^3^.

At the next stage, *Fusarium* strains that showed different variety-specific reactions on the analyzed varieties «Globus» and «Atas» were included in the study. To more accurately assess the degree of aggressiveness of *Fusarium* strains, the study was carried out on eight onion varieties selected by the FSBSI FSVC: «Globus», «Atas», «Sigma», «Myachkovsky», «Boterus», «Tseparius», «Cherny princ» and «Kolobok». Inoculation and recording of the results were carried out using the same method as at the initial stage of the research.

Experimental data analysis and statistical evaluation were performed in Microsoft Excel 2010 and Statistica 10.0. In order to determine the significance of differences in the aggressiveness of *Fusarium* strains, one-way analysis of variance (ANOVA) was used with mean values grouped into homogeneous groups according to Duncan’s multiple range test (*p* ≤ 0.05) [28].

## 3. Results

**Figure 1:**
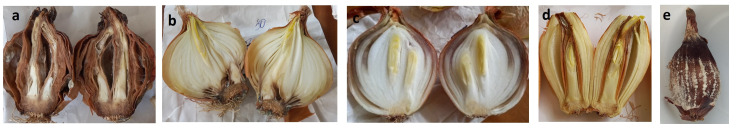
The symptoms of FBR: (**a**)—whole mature bulb; (**b**)—basal plat; (**c**)—neck; (**d**)—central part; (**e**)—seed bulb.

Visually assessed signs of Fusarium rot of bulbs were as follows: localization of symptoms, presence, color and density of sporulation, and consistency and color of the affected tissue. It was found that the disease affected the whole mature bulb in 32% of the cases (Figure 1a). In the remaining cases, only certain parts, such as the basal plat, the neck or the central part, were affected. Half of the analyzed bulbs had onset of the disease on a basal plat, and the progress of the disease was accompanied by darkening of the tissue, maceration of succulent scales and formation of cavities between them. The white or white-pink dense coating of sporulation was observed in some cases (Figure 1b). In most cases, the symptoms of rot in the neck area were observed only when the bulb was cut. These symptoms, consisting of browning and drying of the outer succulent scales, were observed in 13% of diseased bulbs (Figure 1c). Some bulbs had signs of rot in the central part without further spread (Figure 1d). The seed bulbs affected by Fusarium rot were completely dried out with a white coating of sporulation under the outer dry scales (Figure 1e).

**Figure 2:**
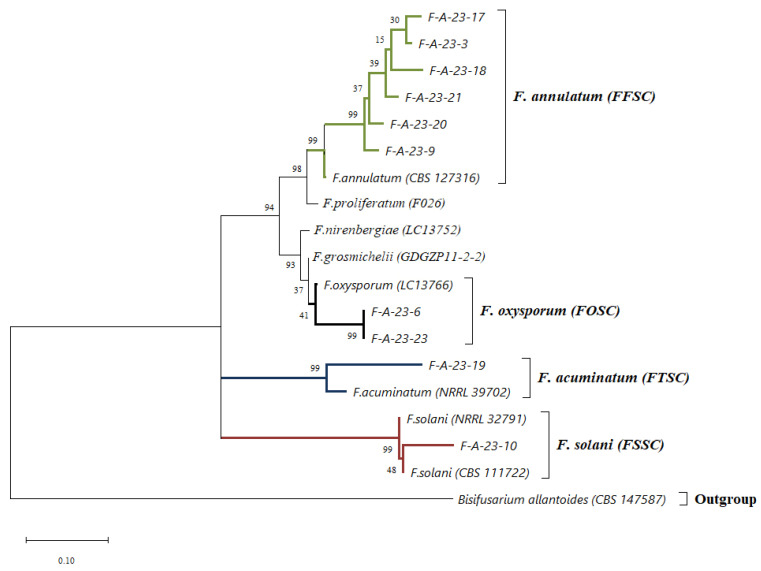
Maximum likelihood phylogenetic tree based on DNA sequence data of ITS, *tef1* and *rpb2* loci of *Fusarium* strains. The bootstrap support values (1000 replicates) are shown at the nodes. The tree was rooted on sequences of *Bisifusarium allantoides* strain CBS 147587.

A total of 20 isolates of *Fusarium* fungi were collected from affected mature bulbs and seed bulbs. After macro- and micro-morphological characteristics, the isolates were divided into groups according to the similarity of microstructures. Isolates with different aggressiveness were selected from each group. There were ten such isolates in total. Species identification of these ten isolates was carried out with molecular method analyzing three genetic loci ITS, *tef1* and *rpb2*. The nucleotide sequences of these loci in the studied isolates were compared with reference sequences from the NCBI database and the supervised FUSARIOID-ID database. The comparison results are presented in the form of a dendrogram (Figure 2). From these data, it is possible to unambiguously identify the species of the studied strains since the sequences of all three analyzed loci on this dendrogram clustered with the reference sequences of the loci of the corresponding species (Figure 2).

Strains F-A-23-3, F-A-23-9, F-A-23-17, F-A-23-18, F-A-23-20 and F-A-23-21 belong to the species *F. annulatum* of *F. fujikuroi* species complex (FFSC); and strains F-A-23-6 and F-A-23-23, based on a combination of molecular and phenotypic characteristics, belong to the species *F. oxysporum* of *F. oxysporum* species complex (FOSC); strain F-A-23-10 belongs to the species *F. solani* of *F. solani* species complex (FSSC); strain F-A-23-19 belongs to the species *F. acuminatum* of *F. tricinctum* species complex (FTSC). Thus, we identified species *F. annulatum*, *F. oxysporum*, *F. acuminatum* and *F. solani* as causative agents of onion Fusarium basal rot in the Moscow Region of the Russian Federation.

The final species attribution of the studied *Fusarium* strains was made relying on the data of both molecular phylogenetic and macro-micromorphological analysis. The morphological characteristics of the identified species are shown in Table 2. Colonies of different species differed in their growth pattern, color, mycelium density and pigment formation. The species *F. annulatum* and *F. oxysporum* produced large numbers of microconidia and a few macroconidia, very similar in appearance. The main difference between these species was the presence of chlamydospores in the mycelium of *F. oxysporum* and their complete absence in *F. annulatum*. It is for this reason that strains F-A-23-6 and F-A-23-23 were eventually identified as *F. oxysporum*. 

Thus, the macro- and micro-morphological characteristics of the studied *Fusarium* strains confirmed their species identity, established by molecular methods.

We further performed a primary evaluation of the pathogenicity of all 20 obtained isolates, consisting of 12 strains of *F. annulatum*, 5 strains of *F. oxysporum*, 2 strains of *F. acuminatum* and 1 strain of *F. solani*. To focus attention on the differences in pathogenicity among fungal strains of the same species, the obtained data for the two studied varieties and replications were averaged (Table 3). The ratio of strains differing in aggressiveness and their locations were different in different *Fusarium* species. Isolates belonging to different pathogenicity classes were identified in the species *F. annulatum* and *F. oxysporum*. Moreover, the largest proportion (33%) of highly aggressive on mature bulbs strains was found in the species *F. annulatum*, and 80% of *F. oxysporum* strains were weakly aggressive. Two strains of *F. acuminatum* were moderately aggressive, and the only strain of *F. solani* isolated from a seed bulb was weakly aggressive for a host plant. Symptoms during natural infection and artificial inoculation of onion scales with all highly aggressive strains of the analyzed *Fusarium* species were identical. Inoculation of succulent scales with weakly pathogenic strains F-A-23-2, F-A-23-1, F-A-23-8, F-A-23-3, F-A-23-6, F-A-23-7, F-A-23-4 and F-A-23-10 virtually did not cause lesion and the pathogen slightly spread beyond the mycelial block, weakly penetrating deep into the tissues of both varieties included in the study. In this group, the volume of the affected area of strains F-A-23-9 (*F. annulatum*) and F-A-23-11 (*F. oxysporum*) was 2–11 times greater than in the case of other weakly aggressive strains, but this difference was nonsignificant (*p* ≤ 0.05).

**Figure 3:**
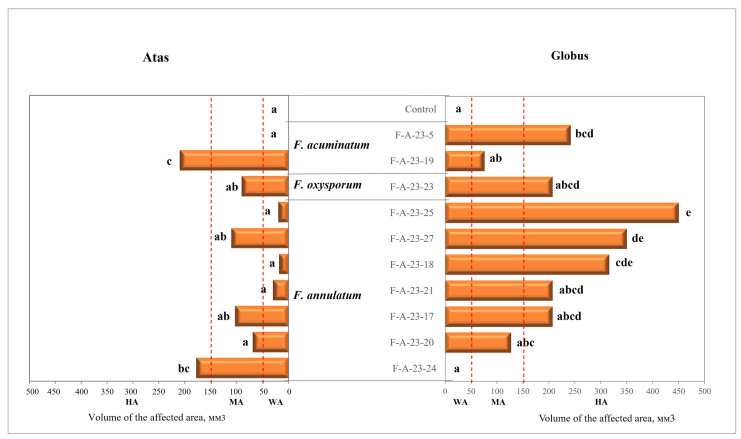
The volume of the affected area of portions of succulent scales of onion bulbs on the tenth day after artificial inoculation with strains of different *Fusarium* species. The values are the mean of five replications of two varieties: «Atas» and «Globus». The combinations of different letters (a–e) next to the bars indicate a significant difference (*p* ≤ 0.05). Designations of aggressiveness classes: WA—weakly aggressive; MA—moderately aggressive; HA—highly aggressive. The boundaries of these aggressiveness classes are indicated by vertical dotted lines.

Moderately and highly aggressive isolates of the species *F. acuminatum*, *F. oxysporum* and *F. annulatum* were used to infect the varieties «Atas» and «Globus». The varieties «Atas» and «Globus» showed different responses to infection by these isolates (Figure 3). Most of the analyzed strains (80%) were weakly aggressive for the variety «Atas». When infected with these strains, either there were no symptoms of the disease, or the volume of the affected area was less than 50 mm^3^. In contrast, strains F-A-23-24 (*F. annulatum*) and F-A-23-19 (*F. acuminatum*) were highly aggressive on the variety «Atas» (Va = 170–201 mm^3^). On the variety «Globus», strain F-A-23-19 (*F. acuminatum*) was weakly aggressive, and strain F-A-23-24 (*F. annulatum*) was non-pathogenic. Seven other analyzed isolates on the variety «Globus» caused clearly defined rot symptoms with the volume of affected area Va ≥ 150 mm^3^. The most aggressive strains were *F. annulatum* strains F-A-23-25 and F-A-23-27 (volume of affected area Va = 207–450 mm^3^, *p* ≤ 0.05). Infection with these strains caused similar symptoms.

Several strains of *F. annulatum*, *F. oxysporum* and *F. acuminatum* were characterized as highly aggressive according to average data. However, these strains were shown to infect two initially studied varieties in a different manner. Therefore, these strains were further used to inoculate another eight onion varieties for a more accurate and informative assessment of their aggressiveness. As a result, the strains F-A-23-19 and F-A-23-5 of *F. acuminatum* and strain F-A-23-25 of *F. annulatum* were shown to be the most aggressive against all studied varieties. The volume of the affected area for these strains was 189–488 mm^3^ on average (Table 4). Moreover, the strain F-A-23-25 of *F. annulatum* was characterized as highly aggressive during the initial pathogenicity assessment (Table 3 and Table 4). In comparison, the strains F-A-23-19 and F-A-23-5 of *F. acuminatum* showed a tendency to increase the level of aggressiveness compared to the initial assessment of two varieties, which is associated with the inclusion in the study of varieties with different levels of resistance. It was found that when onion bulb scales were inoculated with the strains F-A-23-19 and F-A-23-5 of *F. acuminatum*, a critically large volume of the affected area (≥150 mm^3^) was observed in many studied varieties—62.5 and 50%, respectively. 

As a result, the analyzed varieties were shown to have different resistance to specific *Fusarium* species. Thus, when the variety «Sigma» was infected with all tested strains, the symptoms of bulb damage throughout the entire recording period either did not appear or were insignificant and, therefore, did not differ statistically from the control group (Table 4). Whereas in relation to the variety «Globus», inoculation with all strains caused clearly defined rot symptoms with the volume of the affected area Va = 132–2123 mm^3^ depending on the strain. Moreover, the strain F-A-23-23 of *F. oxysporum* severely affected only this variety. It should be noted that the variety «Globus» was assessed as highly susceptible to Fusarium rot both during the initial assessment of pathogenicity and under conditions of natural infection in the field and during storage. Strains F-A-23-19 and F-A-23-5 (*F. acuminatum*) and strain F-A-23-25 (*F. annulatum*) severely affected the varieties «Cherny princ», «Boterus» and «Kolobok» during artificial infection of the bulbs. The varieties «Myachkovsky», «Tseparius», and «Atas» turned out to be highly susceptible only when infected with strains F-A-23-19 and F-A-23-5 of F*. acuminatum* (Va > 100 mm^3^).

Thus, the outcome of infection when inoculating onions with various strains of micromycetes was influenced by both the analyzed *Fusarium* species and the specific resistance of the varieties. As a result, highly aggressive strains of *Fusarium* species associated with onion basal rot were isolated, and different levels of resistance of the analyzed varieties to several or specific species were shown.

## 4. Discussion

An important element of the integrated system for protecting onions from *Fusarium* basal rot pathogens is the resistance of cultivated varieties and hybrids. But when searching for sources of resistance to *Fusarium*, it is important to know the species identity of isolates obtained from affected plants in a specific growing area since the host plant may be resistant to some *Fusarium* species and susceptible to others. In Russia, there are no data on the exact species identification of *Fusarium* that causes onion basal rot, which makes it difficult to carry out the breeding process for resistance. Some publications report *F. oxysporum* isolated from vegetative perennial onion plants with symptoms of chlorosis and wilting. However, the *Fusarium* species identification method is not described in this publication [29].

Analysis of morphological characteristics and molecular taxonomy based on the sequences of the *tef1*, *rpb2* and ITS loci allowed us to identify four species of causative agents of this disease: *F. annulatum* (FFSC), *F. oxysporum* (FOSC), *F. acuminatum* (FTSC) and *F. solani* (FSSC). It is known that the species composition of the pathocomplex, the ratio of differently aggressive strains within these species, the aggressiveness of the prevalent species in a particular mycocommunity and the level of plant resistance determine the nature and severity of the disease [14]. In this study we showed that the outcome of infection when inoculating onions with various *Fusarium* strains was influenced by both the aggressiveness of differently pathogenic species and the resistance of varieties. It was found that in the pathogenesis of *Fusarium* basal rot on onions, *F. annulatum* (60%) and *F. oxysporum* (25%) were dominant among the total set of analyzed strains.

During the initial assessment, we have shown that the species *F. annulatum* and *F. oxysporum* found on onion bulbs are represented by different classes of isolates in terms of their pathogenicity. This finding is consistent with the data of other researchers [10,30]. The latter is a close relative of the species *F. annulatum*. The species *F. annulatum* was found to have the most isolates highly aggressive for mature bulbs (33%). The species *F. oxysporum* had the most isolates that were weakly aggressive for mature bulbs (80%).

The great economic damage caused by *F. oxysporum* as the causative agent of onion basal rot during the growing season and storage has been reported in numerous publications [7,11]. In our research, the species *F. oxysporum* had a variety-specific reaction in relation to the onion varieties included in the study. Thus, 50% of the analyzed varieties, when inoculated with this species, did not differ statistically from the control group of plants. Only the variety «Globus» was characterized by high susceptibility to *F. oxysporum*.

The species *F. solani* was found to cause *Fusarium* wilt of onion plants at the early stages of their development only [31]. This species does not independently cause onion basal rot at later stages of plant development, occurring only as part of a pathocomplex with other aggressive species [32]. We identified only one strain of *F. solani*, isolated from a seed bulb, which showed weak aggressiveness at artificial infection of mature bulbs.

According to a few reports, the species *F.acuminatum* in some countries was more often isolated from the affected vegetative parts of onion and garlic plants with symptoms of chlorosis, necrosis and rot on the leaf blade, less often with symptoms of *Fusarium* basal rot [33,34,35,36]. When artificially inoculated, the leaf isolate of this species was non-pathogenic on mature bulbs and pathogenic on leaves [37]. In our studies, the strains F-A-23-19 and F-A-23-5 of *F. acuminatum* were isolated from different sources (seed bulbs and mature bulbs) and were the most aggressive. More than 50% of the test varieties included in the study were severely affected by this species, with the average volume of the affected area of 155–2123 mm^3^. Only the variety «Sigma» was relatively resistant to these highly aggressive strains. In this variety, by the end of the experiment, a small lesion appeared on some bulbs, but according to the average volume of the affected area, the experimental plants belonged to the same homogeneous group as the control ones.

Often, species identification of the *Fusarium* genus, especially when working with closely related species, is difficult due to the high similarity of their key morphological structures. 

In our studies, we encountered this problem when identifying the species *F. annulatum*, which in the past was often classified as a closely related species, *F. proliferatum* (both species are representatives of FFSC) [38]. At the same time, the ex-type strain of *F. annulatum* was shown to differ from *F. proliferatum* in the formation of highly curved sporodochial conidia [39]. However, other researchers have shown that this morphological feature is atypical for *F. annulatum* since most isolates of this species actually produce only straight macroconidia [40]. As a result, these species were distinguished only at the genetic level using molecular markers. Since, for a long time, only the species *F. proliferatum* was considered the main causative agent of onion basal rot, we did not find information about the harmfulness of *F. annulatum* for this crop. According to recent data, *F. annulatum* caused symptoms of rot on melons in Spain [41]. In China, this species was found in saffron [42]. In the USA, this species was the causative agent of vascular wilt in grapes [43] and root rot in coniferous tree seedlings [44]. 

For the first time, we demonstrated the high aggressiveness of *F. annulatum* strains for onions and the ability of this species to cause onion basal rot. In our studies, 60% of the strains isolated from the affected bulbs were classified as *F. annulatum*. Moreover, based on a comparison of the nucleotide sequences of marker loci in these strains with reference sequences from the NCBI database, these strains could be classified as *F. proliferatum*, but based on the results of comparison of these nucleotide sequences with reference sequences from the supervised FUSARIOID-ID database, these strains belonged to species *F. annulatum*. The criterion for the final classification of these isolates as *F. annulatum* was that they formed only straight macroconidia, which is consistent with recent data from other researchers [40].

In studying the aggressiveness of *Fusarium* strains for various crops, two varieties contrasting in resistance are often used. As our research has shown, only two varieties are not enough for an objective assessment and a wider range of varieties with varying degrees of resistance is required. Thus, the strain F-A-23-23 of *F. oxysporum*, according to the primary assessment of the two varieties, was highly aggressive. Whereas for a larger number of varieties, it was already characterized as moderately aggressive. On the other hand, in the case of strains F-A-23-19 and F-A-23-5 of *F. acuminatum*, increasing the number of analyzed varieties with different resistance allowed us to characterize these strains as more aggressive.

Our study of the species composition of *Fusarium*, which causes onion basal rot, is important for producers of commercial products not only in the Moscow region of the Russian Federation but also in other regions due to the lack of relevant information. In addition, the data obtained on the species composition will have priority in the programs planned by breeders when creating onion varieties and hybrids with resistance to one or more species of *Fusarium*—the causative agents of onion basal rot.

## 5. Conclusions

Results of the present study shed light on the diversity and pathogenicity of *Fusarium* fungi, causing onion basal rot in the Moscow Region of the Russian Federation. The species composition of *Fusarium* fungi was confirmed by the results of molecular phylogenetic analysis. Four *Fusarium* species belonging to different species complexes were identified. These are *F. annulatum* (FFSC), *F. oxysporum* (FOSC), *F. acuminatum* (FTSC) and *F. solani* (FSSC). The most common species were *F. annulatum* and *F. oxysporum*. The species *F. annulatum* was mainly represented by highly aggressive strains affecting mature bulbs. We show for the first time that the species *F. annulatum* is capable of causing onion basal rot, and this species has highly aggressive strains that cause this disease. We deem that the list of *Fusarium* species associated with onion basal rot in this region is still incomplete and requires further research. The obtained data are of practical value for the development of measures to combat *Fusarium* fungi, which cause onion basal rot in the Moscow Region of the Russian Federation. In addition, data about species composition and aggressive isolates may be used in onion breeding for resistance to Fusarium basal rot.

## Figures and Tables

**Table 1:** Sequences of primers used for amplification.

Primer	Sequence 5′–3′	Product Size (bp)	Tm (°C)	A Source
fRPB2-7cf RPB2-11ar	F: ATGGGYAARCAAGCYATGGG R: GCRTGGATCTTRTCRTCSACC	~900	52	[20]
ITS5 ITS4	F: GGAAGTAAAAGTCGTAACAAGG R:TCCTCCGCTTATTGATATGC	~550	56	[21]
EF-1 EF-2	F:ATGGGTAAGGARGACAAGAC R:GGARGTACCAGTSATCATG	~680	55	[17]

**Table 2:** Cultural and micromorphological features of *Fusarium* strains isolated from onions.

Characteristic	*F. annulatum*	*F. oxysporum*	*F. solani*	*F. acuminatum*
Colony:				
growth rate on PDA, mm/day ± S.E.	10.0 ± 1.8	11.5 ± 2.4	7.1 ± 0.2	7.8 ± 0.1
mycelium	White, dense or loose, uniform	White with pronounced radial growth from the center to the periphery of the colony	White and cream with concentric rings	White, dense, uniform
pigment	cream or purple on day 5	light cream on day 7	cream on day 5	lemon on day 6, pink on the periphery of the colony on day 9
Microconidia:				
size, µm ± S.E.	6.3 ± 0.8 × 2.8 ± 0.5	8.5 ± 0.5 × 3.75 ± 0.5	22.8 ± 1.8 × 6.0 ± 0.3	10.3 ± 0.5 × 3.5 ± 0.3
septation	0	0–1	1	0–1
shape	clavate, oval	oval	oval, slightly curved	curved
Macroconidia:				
size, µm ± S.E.	26.3 ± 1.3 × 4.0 ± 0.3	34.5 ± 5.7 × 4.3 ± 0.5	36.3 ± 3.9 × 5.0 ± 1.3	24.5 ± 5.5 × 3.3 ± 0.5
septation	2–3	3–4	2–4	2–4
shape	almost straight	straight to slightly curved	slightly curved	curved
Chlamydospores:				
size, µm ± S.E.	not formed	8.5 ± 1.0 × 8.5 ± 0.8	8.3 ± 0.5 × 8.6 ± 1.0	not formed
shape	not formed	globose	globose, globose-oval	not formed
abundance	not formed	abundant, single, or in pairs	abundant, single, or in pairs	not formed

**Table 3:** The pathogenicity of *Fusarium* isolates differing in the degree of aggressiveness against mature onion bulbs (day 10).

Strains	Place of Localization		Type	Degree of Aggressiveness ^1^	FBR, Score ^2^
					Average Volume of the Affected Area, mm^3^
			Control		0 ^a^
F-A-23-2	mature bulb	basal plat	*F. annulatum*	WA	2 ^ab^
F-A-23-1	mature bulb	neck	*F. annulatum*	WA	2,5 ^ab^
F-A-23-8	seed bulb	basal plat	*F. annulatum*	WA	3,3 ^ab^
F-A-23-3	mature bulb	neck	*F. annulatum*	WA	11,2 ^ab^
F-A-23-9	seed bulb	basal plat	*F. annulatum*	WA	20,5 ^abc^
F-A-23-6	seed bulb	basal plat	*F. oxysporum*	WA	2,5 ^ab^
F-A-23-7	seed bulb	basal plat	*F. oxysporum*	WA	6,5 ^ab^
F-A-23-4	mature bulb	neck	*F. oxysporum*	WA	11,5 ^ab^
F-A-23-11	seed bulb	central part	*F. oxysporum*	WA	23,5 ^abc^
F-A-23-10	seed bulb	basal plat	*F. solani*	WA	8,5 ^ab^
F-A-23-24	mature bulb	whole bulb	*F. annulatum*	MA	88,5 ^abcd^
F-A-23-20	mature bulb	whole bulb	*F. annulatum*	MA	98 ^abcd^
F-A-23-21	mature bulb	neck	*F. annulatum*	MA	118,5 ^abcd^
F-A-23-5	seed bulb	whole bulb	*F. acuminatum*	MA	121 ^abcd^
F-A-23-19	mature bulb	basal plat	*F. acuminatum*	MA	142,5 ^abcd^
F-A-23-23	mature bulb	basal plat	*F. oxysporum*	HA	152 ^bcd^
F-A-23-17	mature bulb	basal plat	*F. annulatum*	HA	155 ^bcd^
F-A-23-18	mature bulb	neck	*F. annulatum*	HA	167 ^cd^
F-A-23-27	mature bulb	whole bulb	*F. annulatum*	HA	230 ^d^
F-A-23-25	mature bulb	whole bulb	*F. annulatum*	HA	235 ^d^

Note: ^1^ WA—weakly aggressive; MA—moderately aggressive; HA—highly aggressive. ^2^ The table shows the average values of the volume of the affected area for two varieties of onion, «Globus» and «Atas», when the succulent scales of the bulbs are infected with an isolate; ^a–d^: values with the same letter do not differ significantly with a probability of 95% according to the Duncan test.

**Table 4:** Aggressiveness of *Fusarium* isolates against mature bulbs of various onion varieties.

Variety	Control	Strains			
		F-A-23-23 *F. oxysporum*	F-A-23-25 *F. annulatum*	F-A-23-19 *F. acuminatum*	F-A-23-5 *F. acuminatum*
Sigma	0 ^a^	0 ^a^	0 ^a^	52 ^a^	77 ^a^
Myachkovsky	0 ^a^	84 ^ab^	59 ^a^	284 ^abc^	126 ^ab^
Cherny princ	0 ^a^	42 ^a^	591 ^c^	826 ^bc^	127 ^ab^
Boterus	0 ^a^	63 ^ab^	325 ^bc^	268 ^abc^	178 ^ab^
Tseparius	0 ^a^	15 ^a^	61 ^a^	105 ^abc^	208 ^ab^
Atas	0 ^a^	35 ^a^	74 ^a^	537 ^c^	129 ^ab^
Kolobok	0 ^a^	86 ^ab^	277 ^abc^	110 ^abc^	942 ^bc^
Globus	0 ^a^	233 ^b^	132 ^ab^	155 ^abc^	2123 ^c^
Average volume of the affected area, mm^3^	0 ^a^	69 ^a^	189 ^abc^	292 ^bc^	488 ^c^

Note: The table shows the average values of the volume of the affected area for eight varieties of onions when the juicy scales of the bulbs are infected with isolates; ^a–c^: values with the same letter do not differ significantly with a 95% probability according to the Duncan test.

## Data Availability

The original contributions presented in the study are included in the article, further inquiries can be directed to the corresponding author/s.
